# Feeding of Nursing Children

**Published:** 1881-03

**Authors:** Ephram Cutter

**Affiliations:** Boston, Mass.


### ARTICLE IV.

### *Feeding of Nursing Children.*

BY EPHRAM CUTTER, M. D., BOSTON, MASS.

In the May No., 1880, of this journal, Dr. Wiley gives an important practical article on infant feeding. On page 132, he pertinently says: "How much suffering would be avoided, how much medicine saved, how many lives spared, if children, from infancy to childhood, could have special attention paid to their diet."

I have always thought that the trouble was generally in the deficient lactation or functional derangement of the mamary gland. I have elsewhere (*American Journal of Obstetrics*, April, 1878,) in a paper "On Food as a Medicine in Agalaxia," given my ideas as to this subject. Still, to enforce the animus of Dr. Wiley's paper, I may, perhaps, express my opinion that, if man should treat the individuals of his own race as well as he treats his cattle, horses and birds, there would be no difficulty in this respect. It is a matter of history that the æsthetics of the senses govern man in the selection of food. We have naught to say against this—only to suggest that the other views of food, as the chemical, physiological, kinetic, pathologic and therapeutic, should not be ignored. This matter of agalaxia comes under the head of food as a physiologic remedy or promoter of functions. The subject is quite a broad one, and for its fuller development we beg to refer readers to a lecture delivered to the Maryland and District of Columbia Dental Association in September, 1879, and published in their *Transactions*. For our present purpose, it is only necessary to say that the dairymen of this country seem to have solved the problem of getting the full supply of milk that their herds can furnish. Experience has taght them that the food must be ample, in good condition, timely served, and contain all the mineral elements that enter into the constitution of milk. Allowance is made for differences in breeds and in individuals of these breeds; also, the kine must be sheltered properly. The animals have nothing to do but to spend all their nerve forces in the facilitation of the work of the epithelial cells of the mammary glands. They are kept quiet and free from worry and excitement. The dairymen do not consult the love of the beautiful of the animals in the selection of the food for their kine. The animals must eat what is furnished to them or die. I have no doubt, however, that the æsthetics of the animals are gratified, as wholesome food is generally beautiful to the senses. The secret of the feeding of kine to produce a good quantity of milk is, other things being equal, to give *whole* food—that is food that contains all the elements that the Creator has incorporated into the bodies of his creatures. It is a matter of philological interest that *hale*, *whole*, *health* and *holy* are all derived from the same root. In order, then, to make a perfect being, the diet must contain the entire circle of the elements. Fortunately for cattle, horses and birds, their ailments are *whole foods*. That is, they have suffered no diminution in their normal elements.

But how is it with woman ? It is a matter of history that their diet is mainly flour, sugar and starch. In the flour we have an imperfect, unnatural food. It certainly is not a whole food. In milling, for the sake of ensuring whiteness and lightness in the bread, a costly process has been pursued, in which there has been a waste of (Liebig says) three fourths of the normal mineral salts found in wheat. The reason of one is, that the gluten ceils wnich are so beautifully disposed over the periphery of the grain of wheat contain coloring matter, and if retained, cause the bread to be brown or black in hue. We cannot explain why society has ordained that *white* should be the *æsthetic color in bread.* It is a matter of taste, but so it is ; and the gluten cells that contain so much phosphorus and other mineral salts have to be removed to graitfy the behests of society's decision that bread is not good unless it is *white.* To put this more definitely : In Johnson's "*How Crops Grow*," many analyses are found. There it is stated that in one thousand grains of wheat there are 8.2 grains of phosphoric arid ; in flour, 2.1 grains of phosphoric acid.

Now, as milk is one of the most remarkable secretions, in the fact that it contains, or ought to contain, all the elements necessary to build up the body, how can we expect the epithelium of the mammary glands of a woman fed on flour to elaborate good milk if fed on an aliment from which three-fourths of the mineral matter has been withdrawn ? But this she has to do, as a general thing. She has to make, as it were "bricks without straw." Is not this a physiological cruelty to her and her offspring? If dairymen did so with their cows, the society for the protection of cruelty to animals would arrest them. I have no doubt, in my own mind, such is the general use of flour, in the form of bread and other preparations, that the function of lactation is greatly impeded by it. For evidence see below.

The effect of feeding on flour exclusively was shown by Magendie years ago. He found that dogs fed on flour died in forty days; on no food they died in the same time; while dogs fed on wheat thrived.

Judge Abbott, of Boston, tells of some sailors wrecked at sea and obliged to live on flour alone. The effect was disastrous for them.

*Sugar.*—This is made up of carbon, hydrogen and oxygen—three elements. It forms a large moiety of the food of man simply because its sweet taste is so agreeable. To be sure it is found in milk, but it would be hard work for the epithelium of the mammary gland to elaborate milk from sugar simply, because milk has over sixteen elements. The bad reputation of cake comes from too much sugar. I do not think the shortening is harmful.

*Starch*, as such and separated, is much used as food. It also is found in flour; indeed, the trouble is, it is too large a constitutent of flour. It contains the same elements as sugar, and in digestion changes into sugar. Now, starch is a valuable and indispensable moiety of food when it is kept to its normal proportion, but when in excess it is not healthy, as seen in the cases of the dogs and sailors. It makes fat, and as such is good food for lactation. But it must not be in excess. It must have mingled with it enough of mineral elements to supply the chemical necessities of milk. It is supposed that the protoplasm of the epithelial cells elects or selects, if you please, the elements from the blood from which to make the milk. It does not create any new elements. Hence, the necessity of the conditions already sta‘ed—that the food must contain all the elements which the chemist finds in the human body.

Another idea is (one laid down by Dr. Salisbury) "that the animal, to be healthy, must have its natural food." This varies with the animal. Ladies observe this rule for their canaries. Stable-keepers observe this rule with their horses, and dairymen with their kine. But what is the natural food of mankind ? Dr. Salisbury says two-thirds animal and one third vegetable food. I agree with him, and my judgment is formed from experience. If, then, man should eat the food found in nature in the above proportions, we should find health. Some may dissent from this doctrine, but we two witnesses testify that we have found it true. We do not class flour or separated starch and sugar as natural foods, since such are not found in nature.

From this we say that, if a nursing mother is put on animal food two-thirds and vegetable food one-third by bulk, she will have milk enough to supply the natural demands of her offspring. We have repeatedly tested this in practice, and our object now is respectfully to call attention to this every-day but important matter, so that obstetricians may see to it that their deliveries get the food that is their own natural and inalienable right; that lactation may be a normal and blessed process, and that the coming generations may get a good start in the world with nervous systems well fed, strong and capable of endurance.

Another thing—is it right for nursing women to have so much strain put on them in work during and after gestation ? Mares with foal and cows with calf are allowed rest, but how often are women worked night and day close up to confinement, and after going through with severe labors are obliged to drudge again in the cares of a family ? Is it a wonder our milk fails ?

"Illustrative Case.—A Primipara has Twin Sons—Feeds on the Salisbury Plan—Entirely Supports Both—Gains Ten Pounds in the First Six Months.—Miss

V. M. I., of Boston, aged 21 years, married and went to reside in the State of Maine. She weighed ninety-eight pounds. She was a women of fine, rare mind, small in size but symmetrical. At the age of seventeen she had a severe illness with typhoid fever, which left her with an obstinate catarrh, which was characterized by offensive, tough, leathery, nasal dejections. This was cured by the application of fuming acid to the site of the ulceration halfway through the nostril. In the course of her first married year she was delivered of twin sons; labor normal. She lived in a rural district, where neighbors feel an interest in such affairs, and she was visited from near and far by interested dames. It is proper to say that the uniform advice in relation to the "suckling" was *not to suckle them at all*, as it was predicted she could not suckle them long, at least. This advice shows, in general terms, that lactation is not always a success, and that nursing-bottles are in use in Maine. Now, the paternal grandfather of the twins was much interested that they should suckle. He therefore consulted me. I told him the sum of what has been here stated. At his request, I gave a type-writer's printed list of things to eat and not to eat. It included all animal and vegetable foods, excluding flour, starch and sugars. The animal food was to be two-thirds, and the vegetable one-third. This list Mrs. I. received, and went on, sustained and strengthened by her father's influence. It was faithfully carried out. At the expiration of six months, one twin weighed seventeen pounds, and the other weighed eighteen pounds. They derived all their nourishment from their mother's breast. They were the pictures of health, and unfailing sources of unmingled delight to all. I think I never saw more domestic happiness in a family than was here. The children, in place of being considered a nuisance, were, indeed, the greatest blessings and well-springs of joy."

This was a severe test for the diet—the straitest I have known. I may fail in some other like cases, but I have never failed in single births to secure an abundant supply of good milk simply by feeding the mother on the same general principles of dairy feeding.

It is unpleasant for women to be compared to cows. But the non-æsthetic nature of this idea should not be allowed to overbalance the teachings of history where it is of the first importance for infants to have good bones, muscles, nerves and organs with which to fight the battle of life.—*Vir. Med. Monthly.*

